# Pharmacogenomics and Opioid Efficacy in Sickle Cell Disease

**DOI:** 10.3390/medicina62010172

**Published:** 2026-01-15

**Authors:** Rabab H. Elshaikh, Asaad M. Babker, Sanaa Elfatih Hussein, Khalid Abdelsamea Mohamed Ahmed, Ashok Kumar Sah, Ayman Hussein Alfeel

**Affiliations:** 1 Medical Laboratory Sciences Department, College of Health and Applied Sciences, A’ Sharqiyah University, Ibra 400, Oman; 2 Department of Medical Laboratory Sciences, College of Health Sciences, Gulf Medical University, Ajman 4184, United Arab Emirates; azad.88@hotmail.com (A.M.B.);; 3 Department of Clinical Laboratory Sciences, College of Applied Medical Sciences, Jouf University, Sakakah 72388, Saudi Arabia; 4 Department of Medical Laboratory Sciences, Jerash University, Jerash 26150, Jordan; khalid.gu89@gmail.com

**Keywords:** pharmacogenomics, sickle cell disease, opioid efficacy, pain management, genetic variants

## Abstract

The impact of genetic variation in sickle cell patients plays a significant role in opioid therapy individual response and pain management. This review aims to provide a comprehensive overview of the importance of exploring genetic variability and its impact on pain management in patients with sickle cell disease. It also explores opioid therapy variability and opioid Safety. With respect to literature, the polymorphisms in the key metabolic enzymes CYP2D6, UGT2B7, and COMT, as well as variations in the *OPRM1*, are important modifiers of the pharmacokinetics and pharmacodynamics of opioids. Variations in the *COMT* gene can influence how the body manages certain brain chemicals and how pain is experienced, while changes in the *OPRM1* gene can alter how well opioids bind to their receptors. They help determine how opioids are broken down in the body, how well they attach to pain receptors, and how pain is felt by someone with sickle cell disease. Patients with reduced-function and ultra-rapid *CYP2D6* alleles have a modified metabolism of codeine and tramadol, which presents either a reduced analgesic response or a risk for increased toxicity. These observations support the case for the need for tailored opioid prescriptions in a population that is genetically diverse, as well as the risk of not having standardized pain measurement, and the absence of clinical implementation. There remains the risk of unrecognized pharmacogenomics, lack of data, and personalized opioid descriptions persist. Future research should focus on integrating genetic testing into clinical practice to optimize opioid selection, personalize medicine, minimize adverse effects, and ensure each patient receives treatment that is both effective and safe to enhance quality of life for individuals with sickle cell disease.

## 1. Introduction

Sickle cell disease (SCD) is a genetic disorder of red blood cells. The disorder occurs because of an abnormal type of hemoglobin known as hemoglobin S. This abnormality leads to the red blood cells becoming misshapen, rigid, and destroyed before the end of their normal lifespan. This leads to organ dysfunction, progressive damage, and chronic anemia through the constant destruction of red blood cells. The condition poses a major global health issue affecting majority of individuals in the world, with an exceptionally high prevalence found in sub-Saharan Africa, the Middle East, and the Americas [1,2].

Vaso-occlusive episodes are the chief cause of morbidity, hospital use, and reduced quality of life for people with sickle cell disease due to their different symptoms. The vascular occlusion that results from episodes of small vessel blockage by sickled red blood cells leads to tissue ischemia, inflammation, and, in extreme cases, infarction. Frequently, VOC pain is severe and severely debilitating, often requiring immediate hospital treatment. The repeated occurrence of VOCs causes not only acute pain but also contributes to the development of chronic pain syndromes, thereby complicating long-term pain therapy throughout the lifespan of the affected. Episodes of VOC are associated with a 9.5% rise in the use of opioids [3,4].

Patients suffering from sickle cell disease typically undergo pain management utilizing opioid analgesics, which have affinity for the mu opioid receptor. They are standard treatments used for this condition. Most people who suffer from sickle cell disorder have used or abused opioids, primarily because of chronic pain associated with their condition. Variation in Opioid Use Across Sickle Cell–Related Crises. Pain control using opioid medications is crucial in the treatment of sickle cell disease; the nature and severity of the opioid needed can vary depending on the sickle cell crisis. 1. Vaso-occlusive crisis (VOC) Because of the severe microvascular occlusion causing ischemic pain, Opioids are a crucial first-line therapy. Pain of this severity managed with intravenous opioids, such as hydromorphone and morphine these can be administered either on a fixed schedule or via patient-controlled analgesia (PCA). Opioids are frequently combined with other pain relievers, including NSAIDs and adjuvants, to enhance analgesic efficacy. 2. Aplastic Crisis: An aplastic crisis not considered severe, as it is a short-term consequence of bone marrow suppression. In such cases, opioids not automatically used; they are reserve for patients experiencing additional pain. Clinical management focuses primarily on treating the underlying condition that triggered the crisis and providing supportive care, such as blood transfusions. Hemolytic crisis most gallstones associated with hemolytic crises are painless, and when pain occurs, it is usually mild. Symptoms are more frequently related to jaundice and anemia. Opioids or other narcotics required only when pain coincides with red blood cell destruction due to Vaso-occlusion. Other factors, including associated symptoms and the patient’s mental state, can also influence pain perception. Significant variability exists in pain management strategies, and differences in opioid response often result in inadequate pain control. While certain users experience pain inadequately alleviated at standard doses, others become overly sedate and depressed, along with experiencing decreased breathing rates when they take standard doses. Using home healthcare can limit the necessity of repeated hospital admissions for people suffering from sickle cell disease, who often must be on increasing doses of pain medication. To avoid excessive opioid prescribing, healthcare providers and payers should focus on preventing acute pain crises [3,5,6].

Research now shows that genetic differences have a significant impact on how individuals react to opioid drugs. A substantial role in shaping opioid pharmacodynamics and pharmacokinetics played by genetic variability in influencing the way individuals perceive pain. Individuals vary significantly in their response to opioids. This difference in response caused by a combination of genetic and environmental factors. The genetic component may be responsible for 30–76% of the variation in how well an opioid works, its side effects, and potential for addiction. Individual variations in the genes that code for enzymes involved in the metabolism of opioids and also for proteins which transport opioids and the opioid receptors themselves, and also proteins involved in intracellular signals, can all affect how an individual responds to opioid pain relief and also how side effects are tolerated [7,8,9].

The genetic factors implicated in the variation in opioid response include genes that influence the metabolic pathway of the opioid, leading to inadequate analgesia in those with a poor metabolic capacity or increased risk of toxicity in ultra-rapid metabolizers. A variety of genes modify the effect of opioid analgesics through their action on the μ-opioid receptor, which involves downstream signaling and binding. A range of demographic and disease-related factors, including the patient’s ethnic origin, sex, haemoglobinopathy, complications of disease, and age, can also influence the degree of heterogeneity seen in treatment response and opioid dosage requirements [10,11].

For this highly variable pain condition, the physician usually relies on the usual trial-and-error process in prescribing opioids for SCD-related pain. Patients who do not receive individualized treatment are likely to experience ineffective pain relief and are at greater risk of suffering from side effects and further episodes of pain. While the integration of pharmacogenomics into pain management practices provides a logical method to tailor opioid treatment, selecting the most effective opioid and the right dosage by reference to a patient’s genetic makeup could reduce the necessity for higher doses, reduce side effects, and lead to fewer hospital admissions [12].

Personalizing opioid therapy with the aid of pharmacogenomics offers a vital contribution to pain management for individuals with sickle cell disease, which is both more patient-centered and safer. The objective of this research was to consider how the genetic makeup of individuals with sickle cell disease influences their reactions to opioid drugs. This information may help in the development of more targeted treatments for pain in those afflicted [13].

## 2. Materials and Methods

A comprehensive manual literature review was undertaken to examine how genetic variation may influence an individual’s response to opioid pain-relief medications. The literature search was performed across academic and biomedical databases, including PubMed, Scopus, Google Scholar, Semantic Scholar, and Medline. Research papers published between 2008 and 2025 were included. A combination of controlled vocabulary terms and free-text keywords was employed, including pharmacogenomics, sickle cell disease, opioid efficacy, pain management, and genetic variants. The search yielded 126 publications. Titles and abstracts manually screened to identify the most relevant studies, after which 100 articles were selected for detailed review based on predefined inclusion and exclusion criteria. Included studies involved patients with sickle cell disease and investigated genetic factors influencing opioid response, analgesic effectiveness, tolerability, or pain-related outcomes. Studies excluded if they did not include SCD populations, lacked a pharmacogenomic focus, addressed non-opioid pain management, involved non-human subjects, or were not original research articles.

## 3. Opioids and Non-Opioid Analgesics

Analgesics, commonly referred to as pain-relieving medications, and systematically categorized into two principal classes: opioid and non-opioid agents, each exhibiting distinct mechanisms of action, efficacy, and potential adverse effects.

### 3.1. Non-Opioid Analgesics

Non-steroidal anti-inflammatory drugs (NSAIDs) like ibuprofen and naproxen alleviate pain and inflammation through the inhibition of cyclo-oxygenase (COX) enzymes, thereby diminishing the production of prostaglandins at the site of injury. Paracetamol, also known as acetaminophen, while frequently categorized alongside NSAIDs, exerts its effects within the central nervous system via COX-3 inhibition, and is characterized by a lack of significant anti-inflammatory properties. These pharmacological agents exhibit a ceiling effect, indicating that escalating the dosage beyond a specified threshold does not enhance analgesic efficacy but rather increases the risks associated with toxicity [14,15].

### 3.2. Opioid Analgesics

This category encompasses substances such as morphine, hydromorphone, fentanyl, and codeine, which attain analgesic effects by binding to specific opioid receptors (Mu Opioid Receptor (ORPM1, Delta Opioid Receptor (OPRD1, and Kappa Opioid Receptor (OPRK1), (μ, δ, κ) located in both the central and peripheral nervous systems. Stimulation of the μ-receptor elicits the most potent analgesic response, yet it is concomitantly associated with adverse effects, including sedation, dependency, and respiratory depression. In contrast to non-opioid analgesics, opioids permit more flexible titration of dosages to achieve enhanced pain relief, albeit this flexibility is accompanied by an increased risk of addiction and overdose, the main receptor actions [16].

#### 3.2.1. Mu Opioid Receptors (*OPRM1*)

**Figure 1:**
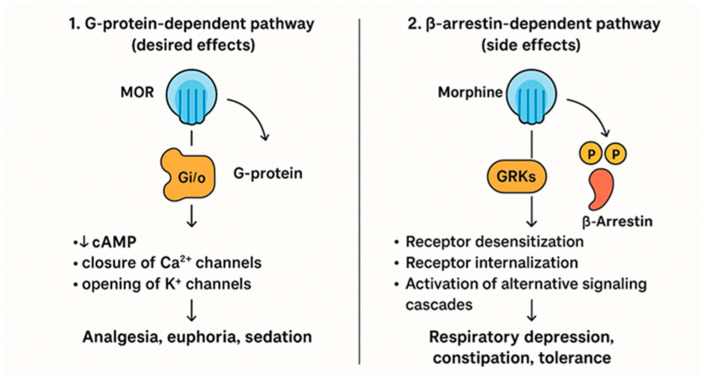
Signaling pathway of Morphine via μ-Opioid Receptor: G-Protein and β-Arrestin Pathways.

**Figure 2:**
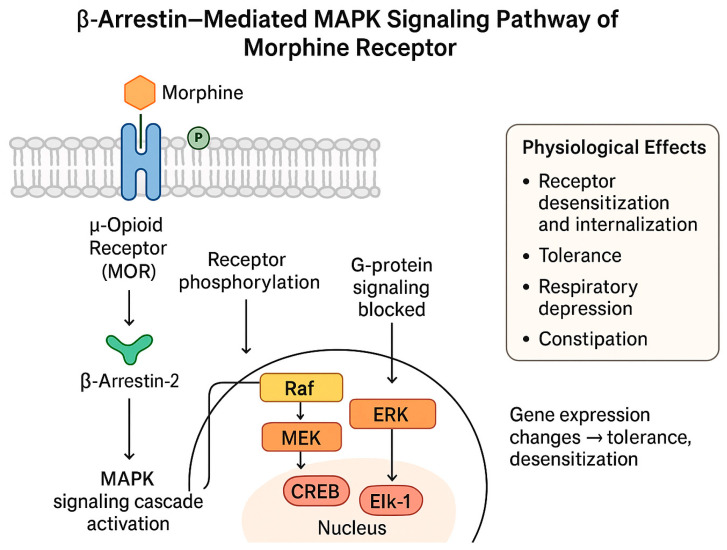
Morphine Mechanism via MAPK signaling pathways.

These are the body’s primary painkillers, found in the brain, spinal cord, and gut, and they respond to natural chemicals like endorphins or to medications like morphine to ease pain and boost pleasure. They are also the reason opioids can be addictive. Over time, the body hides these receptors, making it harder to feel relief without higher doses. Morphine communicates its effects through the μ-opioid receptor, a protein on nerve cells that acts like a switch. When morphine attaches to this receptor, it can trigger two different internal routes. The first is the G-protein pathway, which quiets cellular activity by lowering cAMP levels, reducing calcium entry, and encouraging potassium to leave the cell. These changes calm the neuron and reduce the release of pain-transmitting chemicals, the process that gives morphine its strong pain-relieving action. The second route involves β-arrestin, which steps in after the receptor is activated and tagged by specific kinases. β-arrestin can turn the original signal down, pull the receptor inside the cell, and activate additional signalling networks such as MAPK. This branch is linked to unwanted outcomes, including tolerance, breathing suppression, constipation, and increased sensitivity to pain. The way morphine engages these two pathways’ shapes both its benefits and its side effects [17] (Figure 1 and Figure 2).

#### 3.2.2. Delta Opioid Receptors (OPRD1)

These are emotional stabilizers located in the brain’s decision-making center; they help regulate mood and reduce anxiety. They studied for their potential to protect the brain in conditions like Alzheimer’s, due to their anti-inflammatory actions [17].

#### 3.2.3. Kappa Opioid Receptors (OPRK1)

These receptors are more complex they help with pain but can also trigger feelings of sadness or stress, involved in healing and nerve repair, but when overactive, they may contribute to depression or anxiety [18,19].

#### 3.2.4. Endogenous Pain Relievers

Our body is beautifully equipped to manage pain and stress with its own natural chemicals. When life gets tough, or you push through a workout, it releases endorphins that latch onto mu receptors, easing pain and lifting your spirits. Factors such as stress and exercise can boost the release of these natural pain relievers, enhancing their analgesic effects. Enkephalins, tucked away in your brain and adrenal glands, help quiet the nervous system and block pain signals. Dynorphins interact with kappa receptors and can either soothe or amplify pain depending on the situation. But when medications like morphine, heroin, or fentanyl enter the picture, they mimic these natural helpers with far more intensity. They flood the mu receptors, dull pain, and unleash a wave of dopamine, the brain’s reward signal. Over time, though, your body adapts, making it harder to feel relief without higher doses. That is where addiction can begin. Emergency medications like Naloxone (Narcan) can block these receptors and reverse overdoses, while treatments like Buprenorphine offer a gentler path to recovery. And here is something personal: your genes matter. Few people are naturally more sensitive to opioids or more prone to addiction. One study found that about 14% of people carry genetic combinations that put them at elevated risk, while half fall into a medium-risk zone [20]. Therefore, while pain relief is essential, understanding your body’s chemistry and its vulnerabilities can help you make safer, more informed choices. In short, opioid receptors are part of a powerful system designed to protect and soothe you. But when hijacked by drugs, they can become a source of struggle. Understanding how they work helps us treat pain more wisely and protect mental health along the way. A comprehensive understanding of these pharmacological distinctions is imperative for the formulation of safe and effective pain management paradigms, particularly in chronic conditions such as sickle cell disease (SCD), where the utilization of opioids is prevalent [21,22].

## 4. Opioids in Sickle Cell Disease

The sickle cell condition is a major genetic disorder affecting a considerable number of people. This is primarily due to the unpredictability and severity of the pain, which often results in emergency hospital admissions. In recent years, prescription opioids are considered the most effective method for relieving pain associated with sickle cell disease. In current clinical practice, a combination of treatments is more frequently used. At present, there is evidence to support the use of a combination of opioid and non-opioid pain relievers over using just opioid pain relievers alone. Morbidity and mortality studies recommend the integration of opioids with low doses of NSAIDs or acetaminophen for a limited period. This strategy is used in a way that maximizes its benefits to patients. Using this treatment method can enable people to control their pain more effectively, bring relief from the symptoms of an illness more quickly, and reduce the number of opioid drugs needed. With the current knowledge available, traditional opioid-only treatments for sickle cell disease are no longer in line with best practice and current research [23,24].

In recent years, the U.S. Food and Drug Administration (FDA) has approved multiple non-opioid therapies for managing sickle cell disease (SCD), including hydroxyurea, crizanlizumab, glutamine, and voxelotor. However, opioids remain the mainstay of acute pain management, despite increasing concerns about their long-term effects on neurological pathways. Opioids play a vital role in managing severe Vaso-occlusive episodes; however, prolonged use can lead to tolerance and dependence [25].

### 4.1. Morphine

A complete μ-receptor agonist; it undergoes metabolism via UGT2B7, yielding both active (M6G) and inactive (M3G) metabolites. Genetic polymorphisms in UGT2B7 or *OPRM1* (A118G) may significantly influence drug metabolism and individual patient responses.

### 4.2. Hydromorphone

A potent μ-agonist analogous to morphine primarily metabolized through glucuronidation and less affected by *CYP2D6*, thus providing more consistent pharmacokinetic profiles.

### 4.3. Fentanyl (And Its Analogs)

Exceptionally potent μ-agonists with high lipid solubility facilitating rapid central nervous system penetration metabolized by CYP3A4, rendering them appropriate for patients exhibiting altered *CYP2D6* activity.

### 4.4. Codeine, Oxycodone, and Hydrocodone

These agents classified as prodrugs that necessitate activation through *CYP2D6*. Genetic variability has a profound impact on their therapeutic efficacy individuals classified as poor metabolizers may experience inadequate analgesia, whereas ultrarapid metabolizers are at risk for toxicity. By amalgamating pharmacogenetic knowledge with clinical evaluation, healthcare practitioners can customize analgesic regimens to optimize pain control while ensuring safety for individuals afflicted with sickle cell disease [26].

### 4.5. Methadone and Tramadol

Methadone functions as a potent μ-opioid receptor agonist and simultaneously inhibits NMDA receptors, thus conferring both analgesic and anti-hyperalgesia properties. Conversely, tramadol serves as a less potent μ-agonist that operates through the inhibition of serotonin and norepinephrine reuptake, thereby enhancing analgesic efficacy via dual mechanisms. The metabolic pathways of both substances are intricate, involving the enzymes CYP3A4, CYP2D6, and CYP2B6, in addition to various transport proteins. Genetic polymorphisms within these enzymatic systems can markedly affect the processing of these pharmacological agents in individuals, altering drug concentrations, duration of effect, and the propensity for adverse reactions. Importantly, tramadol is associated with an increased risk of seizure incidents among specific genetic profiles or at heightened dosage levels [5,25].

## 5. Genetic Variations Influencing Opioid Response

Genetic variations exert a considerable influence on the efficacy of opioids and the overall experience of pain management among individuals diagnosed with sickle cell anemia (SCA). Certain individuals have reported insufficient pain relief attributable to mutations in genes, which have implications for the pharmacodynamics of opioids [27]. By facilitating tailored opioid prescriptions grounded in genetic profiles, patient safety is markedly enhanced. To avert the prescription of opioids to patients harboring genotypes with multiple polymorphisms, a particular study formulated a clinical decision support system. Also, the diverse responses to hydroxyurea, a commonly employed therapeutic agent for SCA, modulated by genetic attributes, indicating that genetic testing could augment pain management strategies. Nonetheless, it is essential to acknowledge that contextual factors and individual patient experiences serve as equally critical predictors of treatment efficacy, despite the substantial influence of genetic variability on opioid effectiveness. Consequently, additional research by Gammal 2016 [12] warrants integrating these factors into holistic pain management frameworks tailored for SCA patients.

Acknowledging the role of genetic factors is critical as healthcare professionals explore strategies to individualize pain management for patients suffering from SCA. Genetic Variations in Opioid Response.

## 6. Findings Across Studies

### Patterns of Genetic Assessment and Clinical Effects

Reviewed studies have observed certain recurring patterns in both clinical and genetic studies in relation to the use of opioid treatment in sickle cell disease patients. Research has concentrated primarily on genes that affect the breakdown of opioids, the functioning of opioid receptors, and the transport of opioids. To make sense of the different rates at which people metabolize the drug, researchers also used a categorization of metabolizer types as being ultra-rapid, normal, intermediate, or poor. From a clinical viewpoint, there has been a considerable amount of research concerning variability in how patients react to opioids that are prescribed. Individuals observed a reduction in the pain-relieving properties of analgesics in their case; certain opioids showed no analgesic effect. The problem with opioid treatment is that certain people may be more susceptible to the medication’s side effects or overdose. This patient population exhibited variability in opioid sensitivity and varied opioid dose requirements; this variability was further evidence of the heterogeneity of opioid response in such patients. Research has revealed significant variability in the results of pain treatment, suggesting the need for customized pain relief plans. The considerable diversity observed in various metabolizer phenotypes or genetic variants among patient populations indicates that a one-size-fits-all approach is not suitable for the treatment of sickle cell disease with opioids. Studies have observed certain recurring patterns in both clinical and genetic studies in relation to the use of opioid treatment in sickle cell disease patients. Research has concentrated primarily on genes that affect the breakdown of opioids, the functioning of opioid receptors, and the transport of opioids. To make sense of the different rates at which people metabolize the drug, researchers also used a categorization of metabolizer types as being ultra-rapid, normal, intermediate, or poor. From a clinical viewpoint, there has been a considerable amount of research concerning variability in how patients react to opioids that are prescribed. Considerable numbers of individuals observed a reduction in the pain-relieving properties of analgesics in their case; certain opioids showed no analgesic effect. The problem with opioid treatment is that certain people may be more susceptible to the medication’s side effects or overdose. This patient population exhibited variability in opioid sensitivity and varied opioid dose requirements; this variability was further evidence of the heterogeneity of opioid response in such patients. Research has revealed significant variability in the results of pain treatment, suggesting the need for customized pain relief plans. The considerable diversity observed in various metabolizer phenotypes or genetic variants among patient populations indicates that a one-size-fits-all approach is not suitable for the treatment of sickle cell disease with opioids (Table 1).

## 7. Thematic Analysis and Discussion

Research into the pharmacogenomics of sickle cell disease has revealed that differing reactions to painkillers attributed to variations in genetic makeup that affect the body’s response to analgesics. Individual differences in the way people metabolize opioids along with the way they move from the bloodstream into the brain and how they interact with pain receptors and pain perceptions influence both opioid effectiveness and their potential for harm. These findings suggest that a variety of genes contribute to the opioid response; one key gene is *CYP2D6*, which is directly relevant to the prescribing clinician, whereas additional genetic factors affect the severity of pain, the amount of opioid required and the tolerance that develops to opioid side effects [27,28,29,30,32,37].

### 7.1. Genetic Variations and Allele Frequencies

#### 7.1.1. Opioid Receptor Genes

The main opioid receptor involved with most opioids used in medicine is the mu opioid receptor. This receptor activated by multiple drugs which mimic the effect of the body’s own pain killing substances. Variations in the *OPRM1* gene can either affect how much of the opioid receptor expressed in cells or how well the opioid molecules bind to the receptor. This could lead to differences in the sensitivity to opioids. This variant, A118G (rs1799971), has produced disparate analgesic responses in patients with sickle cell disease, resulting in certain patients requiring higher doses of morphine to achieve pain relief. Conversely, others exhibit neither a change nor an increased sensitivity [27,38,39,40].

#### 7.1.2. Cytochrome P450 Enzymes

Individuals with certain genotypes of the *CYP2D6* gene, responsible for metabolizing the pain relievers codeine and hydrocodone, may process these in their liver at varying rates. In a pediatric SCA population, almost 37.5% displayed normal metabolism, whereas about 5.3% were metabolically slow. This could affect the efficiency of analgesics used in the treatment. Oxidation of certain opioids is primarily through CYP enzymes. As an example, tramadol and codeine need the CYP2D6 enzyme to metabolize to their active compounds, morphine and O-desmethyltramadol, respectively. Both fentanyl and oxycodone are oxidatively metabolized in the liver by the cytochrome P450 enzyme system specifically by the CYP3A4 isoenzyme. This phase may either activate pro-drugs or inactivate active drugs thus affecting the overall pharmacological effect. Metabolism of opioid drugs crucially influenced by CYP3A4 and CYP2D6 enzymes. This gene is very variable in its expression regarding the metabolism of drugs, and its expression classified as poor, average/normal/extensive or ultrarapid metabolizer. Genetic variations in drug metabolizing enzymes may lead to either insufficient or excessive effect of analgesics. In certain cases, the effect of certain analgesics significantly may reduce in certain individuals. The efficacy of opioid therapy in sickle cell disease patients varies with the genetic makeup of the patient, specifically with the *CYP2D6* gene. Patients who have a genotype that does not produce as much of the enzyme that metabolizes morphine have experienced increased pain episodes [12,37]. While less common, variations in the *CYP3A4* gene could affect how the body processes opioid medications such as fentanyl and oxycodone, with an impact on their removal from the body and exposure levels in the blood [25,28].

#### 7.1.3. COMT and ABCB1 Genes

The enzyme which produced by the *COMT* gene breaks down the chemicals, known as catecholamines, that the body uses in pain perception. The Val158Met polymorphism influences the activity of the enzyme and thereby influences opioid and pain thresholds. People who have low activity of the COMT enzyme experience heightened pain sensitivity. Those with the Met/Met COMT genotype have a greater incidence of hospital treatment for short-term conditions compared to those with the Val/Val genotype. Research found another genetic factor contributing to the severity and variability of chronic pain in those with sickle cell disease—the β2-adrenoreceptor [33,34].

Opioids transported by proteins such as P glycoprotein, a product of the *ABCB1* gene, which plays a significant role in the distribution of the drug in the body. The cells themselves can release the opioid, including the transport of the substance across the blood–brain barrier. This affects the concentration of the drug at its target location. A protein known as P glycoprotein, which encoded by the gene *ABCB1* or *MDR1*, participates in opioid transport across the blood–brain barrier. Individuals carrying the C3435T variant in the gene may experience reduced penetration of opioid pain relief into the central nervous system. This phenomenon leads to diminished pain relief efficacy and the need for increased doses [27,32,41,42].

#### 7.1.4. UGT2B7 and Inflammatory Genes

The *UGT2B7* gene recognized as demonstrating a correlation with pain in individuals with SCA and implicated in opioid metabolism [31]. In determining the most effective pain management treatment with fewer side effects, as discussed by Gehling in 2023 and M. Yee in 2013, genetic polymorphisms can have a considerable impact on the prescribing of opioid medication [8,43].

Variants of the *UGT2B7* gene connected to increased sensitivity to pain, through the influence on the metabolic processes of morphine and its transport and sensitivity to pain. Glucuronic acid is an example of a polar molecule that attaches itself to the metabolites of the opioid, thereby increasing its water solubility for excretion. The drug morphine converted by the body into an inactive compound, morphine-3-glucuronide, as well as an active one, morphine-6-glucuronide, through a process called glucuronidation [27]. In a Zimbabwean study, a *UGT2B7* gene variant (rs73823859) was associated with increased levels of pain. The prevalence of this gene variant along with the *CYP2D6* variant is high among African and African American populations. It is thus crucial to take into consideration the genetic makeup of an individual’s population when prescribing opioids for people with sickle cell disease [11,31,44] (Table 1 and Table 2). 

### 7.2. Metabolic Phenotypes and Clinical Implications

The unique chemical profiles that result from variations in genes which affect the metabolism of drugs including narcotics termed metabolic phenotypes. In sickle cell disease, various clinical signs and symptoms have particular importance. Pain control for such sufferers often proves challenging due to the drugs’ differing effects on each patient. These differences can enable healthcare providers to predict drug reactions and treatment outcomes, which improves the treatment. The CYP2D6 enzyme has a considerable influence on the metabolism of multiple types of opioids, amongst them tramadol and codeine. Individual variations in the *CYP2D6* gene can lead to varying degrees of metabolism of analgesics and may result in clinically significant variations in effect. People with this metabolism pathway take a longer time to convert codeine into its active substance. This may result in inadequate pain relief from the medication. certain patients have an extremely high rate of metabolism for these drugs. This can result in higher peak concentrations which increase the risk of these drugs’ side effects. Children who have a genetic variation in the *CYP2D6* gene, which results in reduced enzyme activity, are at a higher risk of codeine’s failure to provide pain relief. In addition, individuals homozygous for these alleles (*17, *29, or *41) have a decreased ability to synthesize morphine from codeine and as a result suffer more frequent episodes of Vaso-occlusive crisis necessitating hospital admission. People with undetectable levels of morphine in their blood admitted to acute care, showing the impact of the *CYP2D6* gene on how well morphine works and its safety. The CYP3A4 enzyme is also involved in metabolizing a subset of opioids, such as fentanyl and oxycodone. Individual variations in the *CYP3A4* gene can result in significant differences in the rates at which different people metabolize drugs and, in turn, can influence the potential therapeutic benefits and potential side effects. While CYP3A4 genetic variability studied less in sickle cell disease populations than *CYP2D6*, it adds another layer of complexity in prescribing opioids. Personalized opioid dosing in SCD should start with *CYP2D6* testing before prescribing codeine or tramadol [32,37,45,46] (Table 2).

### 7.3. Additional Genetic Factors Affecting Analgesia

In addition to the impact on drug metabolism and transport, there are genetic factors contributing to inflammation that influence pain severity and the amount of opioid taken by patients with sickle cell disease. Among multiple genetic variations which may influence an individual’s response to pain and their need for opioid, genetic variations in cytokines IL6, IL1 alpha and TNF-alpha have been particularly significant. They have been associated with higher-than-average levels of chronic pain in children and increased opioid usage as a result. A genetic variation, specifically the IL1A T allele, has been associated with persistent pain syndromes in individuals with sickle cell disease. This indicates the long-term pain experiences in these individuals may shaped by their genetic predisposition towards inflammation [35,36].

### 7.4. Clinical Manifestations of Poor Analgesic Response

A considerable number of patients with sickle cell disease continue to suffer from chronic pain even after treatment with opioid painkillers. This is especially true of episodes known as Vaso-occlusive crises when the pain can be severe and unresponsive to usual doses of painkillers. Variable pain patterns exist in other SCD complications, including aplastic and hemolytic crises, splenic sequestration, and acute chest syndrome. Administration of opioid may be necessary urgently together with non-opioid pain relief medicines in certain cases, by contrast cases managed primarily with supportive treatment and monitoring and the use of strong pain relief medicine for pain that is severe. These findings suggest the value of using personalized treatments. Genetic information on how an individual with sickle cell disease metabolizes opioids in conjunction with a clinical evaluation to ensure the selection of the most appropriate opioid medication and dose. This should reduce the likelihood of the patient suffering adverse effects and improve pain relief [31,32,37,47,48,49].

### 7.5. Other Factors Influencing Opioid Efficacy

Non-genetic factors also influence opioid effectiveness in SCD. Age plays a notable role—pain crises are often more severe in younger patients, while adults may develop opioid tolerance that diminishes response, future research could explore the interplay between genetic and environmental/clinical factors for a more holistic understanding. Concurrent hydroxyurea therapy may further affect outcomes, as reduced-function *CYP2D6* alleles were more common among children receiving hydroxyurea, and codeine treatment failure was more frequent in this group. Variability in hydroxyurea response itself noted as a potential confounder [4,31,32,47,50].

### 7.6. Population-Specific Genetic Insights

A consistent finding across studies is the high prevalence of reduced-function *CYP2D6* alleles in African and African American SCD populations, with significant implications for codeine and tramadol metabolism. The Zimbabwean data [31] extend this evidence to sub-Saharan Africa, highlighting both *CYP2D6* and *UGT2B7* variants as key genetic factors influencing pain outcomes. However, most available data come from pediatric populations in the United States, with limited studies involving adults or African cohorts, emphasizing the need for broader, multi-ethnic research.

## 8. Treatment and Management Approach

When it comes to treating and managing sickle cell disease (SCD), a multifaceted and personalized approach is essential. Recent advances in pharmacogenomics have made pain management more precise, especially for patients with SCD or those dealing with opioid use disorder (OUD). Genetic testing can play a pivotal role in guiding opioid prescriptions by predicting how an individual metabolizes certain drugs, helping reduce the risks of side effects, toxicity, and dependency.

For instance, pharmacogenetic evaluations in SCD patients have shown significant variability in opioid responses. Some individuals are ultrarapid metabolizers, while others are poor metabolizers, requiring careful adjustment of dosage to ensure safety and effectiveness. These insights allow healthcare providers to tailor pain management strategies, optimizing pain relief while minimizing complications from medications. The integration of pharmacogenomics into clinical practice has shown promising outcomes, such as improved quality of life, reduced pain severity, lower opioid consumption, and fewer hospital admissions.

Despite these benefits, widespread implementation remains limited. Current clinical guidelines focus largely on *CYP2D6* and provide minimal guidance on other genetic pathways [26]. Moreover, many healthcare professionals lack the training and expertise necessary to apply pharmacogenomic insights effectively. Efforts are underway to integrate genetic information into electronic health records and decision-support systems to address these gaps. Within SCD care, research is moving toward customizing opioid therapy according to each patient’s genetic profile. Genetic markers associated with opioid efficacy, safety, and dependence suggest that routine genotyping could improve outcomes during Vaso-occlusive episodes, reduce the risk of misuse, and decrease hospitalization rates. However, translating these findings into everyday practice, especially for paediatric and ethnically diverse populations, remains a challenge [39,51,52].

Beyond SCD, pharmacogenomics holds great potential in addressing the opioid crisis through genetic risk assessments, individualized dosing, and structured tapering strategies. Key gene polymorphisms—such as *CYP2D6*, *OPRM1*, and *UGT2B7*—significantly influence opioid metabolism and response, emphasizing the value of personalized pain management. As a cornerstone of precision medicine, pharmacogenomics aims to enhance analgesic efficacy, reduce adverse effects, and prevent addiction. Its successful adoption into routine practice will require further validation studies, standardized testing protocols, and comprehensive clinician education [38,53,54].

SCD causes various types of pain, each requiring a different management approach. Vaso-occlusive crises, the most common and painful manifestation, are typically managed with rapid administration of opioids, often via patient-controlled analgesia (PCA), alongside non-opioid pain relievers. In contrast, aplastic and haemolytic crises stem from sudden drops in haemoglobin and accelerated red blood cell destruction. While these episodes are distressing, treatment priorities include blood transfusions and careful monitoring, with opioids used only for severe pain.

In children, splenic sequestration can cause sudden spleen enlargement and changes in blood flow. Here, pain management must be individualized rather than relying on routine opioid prescriptions. Acute chest syndrome often leads to additional pain, such as back, rib, or chest pain. Excessive opioid use in these cases can depress breathing and reduce oxygen levels, so clinicians must carefully balance effective pain relief with respiratory safety, using the lowest effective opioid dose.

**Figure 3:**
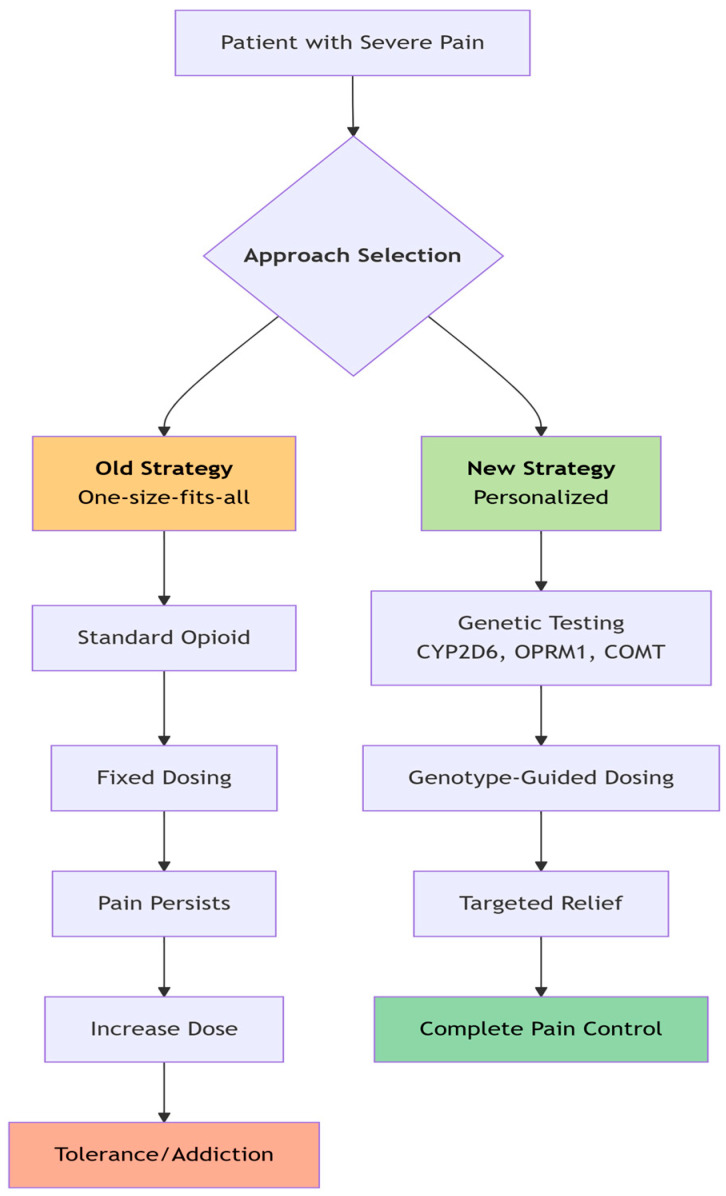
New Strategy for pain management incorporating genetic testing and individualizing medication.

Taken together, this evidence supports a shift from standardized treatment protocols to patient-tailored strategies. Pharmacogenetic screening offers valuable guidance for clinicians, enhancing the effectiveness of pain management while reducing drug toxicity in patients with SCD [46,48,49,54] (Figure 3).

## 9. Implementation Challenges in Personalized Medicine

The way the body metabolizes certain opioids, such as tramadol and codeine, is influenced by a person’s genes. The Clinical Pharmacogenetics Implementation Consortium (CPIC) recommends using a patient’s genetic makeup to guide warfarin dosing. This is important because some individuals metabolize tablets either too quickly or too slowly, leading to inadequate pain relief or adverse side effects. However, many patients with sickle cell disease (SCD) would not benefit from genetic testing, as they often lack a family history of the disorder, making such testing redundant. Most opioids used in SCD pain management, such as hydromorphone or morphine, are unaffected by these types of genetic mutations. Additionally, genetic testing may not always be available, can be extremely expensive, and many hospitals—particularly in less affluent areas—lack the facilities to perform such tests. Using genetic testing selectively, only when there is a clear need, may be more beneficial than incorporating it into standard procedures [48]. Although pharmacogenetic-guided opioid prescribing has been demonstrated to be both feasible and safe, its integration into routine clinical practice remains limited. Major barriers include the absence of standardized pain outcome measures, limited access to genotyping infrastructure, and insufficient incorporation of pharmacogenomics into electronic health systems. Clinician education and the development of clear guidelines are essential to support the interpretation and application of genetic data in routine care. Health care providers and payers should prioritize the prevention of pain crises rather than restricting opioid prescribing [55].

## 10. Research Limitation and Clinical Gaps

Current research remains limited by small sample sizes and a lack of sufficient genetic data. Adjusted medication doses, or differences in hospital policies. Also, the records were not always complete, so we might not have had all the details about how bad the pain was or exactly when medications were given. Plus, we did not have genetic results for everyone, so we could not really connect genes with how people responded to treatment. Keep these things in mind when you look at what we found. 

Only a few studies have effectively integrated pharmacogenomic findings with standardized pain assessment tools or opioid pharmacokinetic outcomes. Despite the available evidence, further research is essential to better understand how genetic, demographic, and clinical factors interact with opioid dosing and pain management in individuals with Sickle Cell Anaemia (SCA). Pain perception is influenced by environmental and psychosocial factors, such as culture and stress, yet genetic variability plays a crucial role in shaping how patients respond to opioid therapy. This highlights the importance of identifying and characterizing genetic variants associated with opioid metabolism [56].

## 11. Conclusions

Pharmacogenomics offers a promising approach to improving pain management in patients with sickle cell disease (SCD), particularly those who need opioids. Genetic variations, especially in *CYP2D6, OPRM1,* and *UGT2B7*, can influence how patients respond to pain medications. Understanding these genetic differences allows healthcare providers to personalize opioid therapy, helping to achieve better pain relief while reducing the risk of side effects, toxicity, and dependence.

Clinical studies have shown that genotype-guided prescribing can reduce pain intensity, decrease hospital visits, and enhance overall quality of life for SCD patients. However, there are still challenges to making pharmacogenomics a routine part of care. These include limited clinical guidelines beyond *CYP2D6*, a lack of standardized testing, insufficient clinician training, and the high cost of genetic testing.

This review identified promising pharmacogenetic markers for opioid therapy, including *CYP2D6, CYP2C8, CYP3A4, CYP2C9, CYP3A4, CYP3A5, DRD2, UGT2B7, ABCB1, ABCC3, CYP2D617, CYP2D629, SLC22A1, OPRM1, COMT,* and *KCNJ6*. Which could help tailor pain management based on individual genetic profiles, further investigation is needed to fully elucidate their complex interactions and translate these findings into actionable clinical guidelines for optimizing pain management in this vulnerable patient population [5,8,12,27,28,29,30,32,37,54] (Table 1).

Integrating pharmacogenomic data into electronic health records and decision-support systems could make its use more practical in clinical settings. Further research in larger and more diverse patient populations, including children, is also important to ensure safe and effective application.

Overall, combining pharmacogenomics with pain management is a key step toward personalized medicine, ensuring each patient receives treatment that is both effective and safe. Continued efforts in research, education, and standardization will be essential to make this approach a regular part of caring for people with sickle cell disease.

In summary, pharmacogenomic variability significantly influences opioid efficacy, safety, and pain outcomes in sickle cell disease. *CYP2D6* genotype is the strongest and most actionable predictor of opioid response, supporting routine pre-emptive testing before codeine or tramadol use. Additional genetic modifiers—affecting drug transport, metabolism, receptor signalling, and pain perception—explain residual variability and justify an integrated pharmacogenomic framework for personalized pain management in SCD.

## 12. Future Directions

Future research should focus on larger and more diverse cohorts of individuals with sickle cell disease (SCD) to elucidate how genetic variation affects opioid response. Expanding the scale and diversity of studies will enhance the identification of genetic patterns, supporting the development of truly individualized pain management strategies.

Integrating pharmacogenomic testing into routine clinical practice is critical, enabling clinicians to tailor opioid therapy to each patient’s genetic profile. Linking genetic data with clinical outcomes—including pain severity, drug levels, side effects, and overall patient well-being—will ensure that findings translate into meaningful improvements in care.

As pain is influenced by more than biology, future studies should examine the interplay between genetics and environmental factors such as stress, cultural background, and life circumstances, providing a holistic understanding of variable treatment responses. Comparative trials evaluating genetically guided prescribing versus standard care are warranted to determine whether personalized approaches improve safety and efficacy. Special attention should be given to adult populations, who remain underrepresented in many SCD studies.

By addressing these priorities, future research can advance precision, effectiveness, and patient-centeredness in pain management for individuals living with SCD.

## Figures and Tables

**Table 1:** Key Genetic Polymorphisms Influencing Opioid Response in Sickle Cell Disease. →: lead to or Clinical effect.

Gene/Variant	Opioids Affected	Functional Role	Observed Clinical Effects in SCD	Clinical Implications	References
*CYP2D6* (*2, *4, *5, *10, *17, *29, *41)	Codeine, Tramadol, Hydrocodone, Oxycodone	Metabolizes prodrugs to active forms	Poor metabolizers→reduced analgesia; Ultrarapid→toxicity; high prevalence of reduced-function alleles in African populations	Preemptive genotyping to guide opioid selection and dosing	[8,28,29,30,31]
CYP3A4 (*1G)	Fentanyl, Oxycodone, Methadone	Opioid metabolism	Reduced clearance: lower dose requirements observed	Potential for dose adjustment; further SCD-specific PK studies needed	[27,28,29,32]
UGT2B7 (rs7438135, rs62296959, rs73823859)	Morphine, Codeine	Glucuronidation to active/inactive metabolites	Influences morphine efficacy, pain intensity, and side effects	Genotype-guided dosing may optimize analgesia and reduce adverse effects	[27,31,32]
ABCB1 (MDR1) (C3435T)	Morphine, Fentanyl, Methadone	P-glycoprotein efflux at BBB	T allele→higher opioid requirements, reduced CNS penetration	May predict dose needs and treatment resistance	[5,27,32,33,34]
*OPRM1* (A118G)	Morphine, Fentanyl, Oxycodone	μ-Opioid receptor binding and signaling	G allele→reduced potency, higher dose needs; mixed evidence in SCD	May inform dose adjustments in non-responders	[3,5,27,32,33]
COMT (Val158Met, rs4633)	Morphine, Oxycodone	Catecholamine metabolism; modulates pain	Met allele→increased pain sensitivity, higher acute care utilization	May guide personalized dosing, consider gender-specific effects	[5,27,32,33,34]
IL1A/IL-6/TNFα (rs1800587, rs1800797, rs1800629)	Indirectly affects opioid response	Inflammatory cytokines modulate pain and opioid sensitivity	T allele (IL1A)→higher baseline pain, chronic pain risk	Potential biomarkers for opioid-sparing strategies	[35,36]

**Table 2:** Metabolic Phenotypes and Clinical Implications of Key Pharmacogenetics in Sickle Cell Disease.

Gene	Metabolizer Phenotype	Effect on Opioids	Clinical Outcome in SCD	Recommendation
***CYP2D6*** [8,28,29,30,31]	Poor Metabolizer	Codeine/tramadol not activated	Inadequate analgesia; ↑ hospital visits	Avoid codeine/tramadol
	Ultrarapid Metabolizer	Rapid activation→high morphine levels	Toxicity risk (overdose, respiratory depression)	Avoid codeine/tramadol
	Normal Metabolizer	Standard activation	Expected efficacy with standard dosing	Standard dosing acceptable
***CYP3A4*** [12,25,28,37]	Reduced Metabolizer	Slowed breakdown of fentanyl/oxycodone	Prolonged effect; toxicity	Lower starting dose; monitor
	Normal Metabolizer	Normal clearance	Standard efficacy/safety	Standard dosing
***UGT2B7*** [27,31,32]	Reduced Activity	Less morphine→active metabolite (M6G)	Poorer pain control; variable side effects	Consider dose adjustment or alternative opioid
	Increased Activity	More active metabolite	Better analgesia; side effects	Standard or lower dose
***ABCB1*** [5,27,32]	Reduced Efflux (T allele)	More opioid enters brain	Better analgesia: lower dose needed	May require dose reduction
	Increased Efflux (C allele)	Less opioid enters brain	Poor analgesia: higher dose needed	May require dose increase

## Data Availability

Available upon request from the corresponding author.
